# PP04 Assessing The Utility Of Natural Language Processing In Generating A Granular Estimated Indication For A Horizon Scanning Database

**DOI:** 10.1017/S0266462324001788

**Published:** 2025-01-07

**Authors:** Caitríona Ní Choitir, Marie Harte, Daisy Duell, Irina Odnoletkova, Anna Bergkvist Christensen, Marie Persson, Helle Bräuner, Marcus Guardian, Alzbeta Alzbeta Tuckova, Brian Wilkinson, Beth Kuzmak, Roisin Adams, John Shaw, Craig Boyce, Prashanth Palakollu, Eileen Erinoff

## Abstract

**Introduction:**

Detailed, precise information on a pharmaceutical’s projected therapeutic use is required for horizon scanning. Inferring an estimated indication from trial protocols is a key skill of horizon scanners. The International Horizon Scanning Initiative (IHSI) database utilizes semi-automated data collection. This pilot aimed to verify that the extraction of relevant word sets to generate an estimated indication could be semi-automated.

**Methods:**

Ten drugs approved in Europe in 2021 were selected as the pilot test set. The test set included drugs approved for the treatment of rare diseases (n=4), haemato-oncology (n=3), and non-oncology conditions (n=3). Eight of the drugs were approved based on phase III trials. The assessment comprised a review of the pivotal trial that supported product registration for these drugs. We undertook a comparison between a human curator and a natural language processing (NLP) algorithm in generating granular tags relating to key aspects of the drugs’ estimated indication (stage of disease, patient-specific subgroup, and place in treatment).

**Results:**

In 50 percent of cases, the NLP accurately tagged a word or word set related to stage of disease, patient-specific subgroup, or place in treatment, which was also tagged by human curators. In 50 percent of cases, the NLP did not identify words or word sets tagged by human curators. Where relevant, the NLP successfully tagged the same word sets relating to stage of disease for all drugs in the test set. The same word sets relating to patient-specific subgroup were successfully tagged for three drugs in the set. NLP successfully tagged word sets relating to place in treatment for two drugs.

**Conclusions:**

The NLP algorithm is successful in extracting relevant word sets, which can be used to generate an estimated indication in an automated or semi-automated process. The pilot highlighted that further testing is required to advance the sensitivity of the algorithm. Further piloting exploring both unsupervised and supervised modeling approaches (named entity recognition and deep neural networks, respectively) is planned.

